# Exploring contextual barriers and facilitators to sustaining mental health integration in primary care: a mixed-methods analysis of adaptive mechanisms and multi-level dynamics in Lagos, Nigeria

**DOI:** 10.7189/jogh.15.04305

**Published:** 2025-11-07

**Authors:** Abiodun O Adewuya, Bolanle Ola, Seye Abimbola, Jibril Abdulmalik

**Affiliations:** 1 Lagos State University College of Medicine, Department of Behavioral Medicine, Ikeja, Lagos, Nigeria; 2 Centre for Mental Health Research and Initiative, Public Health Research Unit, Ikeja, Lagos, Nigeria; 3 The University of Sydney, School of Public Health, Sydney, Australia; 4 University of Ibadan, Department of Psychiatry, Ibadan, Nigeria

## Abstract

**Background:**

Mental health interventions in low- and middle-income countries (LMICs) face significant sustainability challenges, often leading to ‘programme drift’ (protocol deviation ) and ‘voltage drop’ (reduced effectiveness). While implementation science frameworks emphasise fidelity, they often fail to explain how frontline providers in resource-constrained settings maintain services. Here, we investigate how adaptive mechanisms function as legitimate sustainability strategies within Lagos, Nigeria's Mental Health in Primary Care programme, which contends with chronic underfunding, high staff turnover, and community stigma.

**Methods:**

We conducted a convergent mixed-methods study in six Lagos local government areas. Data were collected from 130 stakeholders (policymakers, managers, health workers, care recipients) through quantitative surveys and from a nested subsample of 70 participants through in-depth interviews and institutional ethnography. We analysed quantitative data using multiple regression and qualitative data using thematic analysis, systematically integrating the findings through triangulation to produce meta-inferences about sustainability dynamics.

**Results:**

Systemic constraints, particularly underfunding (<2% of health budget) and high staff turnover (30% annually), drove programme drift and community stigma, deterring 40% of patients and contributing to voltage drop. However, this drift often manifested through constructive adaptive mechanisms, including informal peer mentoring networks and role flexibility, which maintained service continuity. Multiple regression (R^2^ = 0.45) identified leadership (*β* = 0.42), infrastructure (*β* = −0.35), and stigma (*β* = −0.30) as significant predictors of sustainability. Mixed-methods integration revealed these adaptations were the primary mechanism through which effective leadership operated – a dynamic invisible to quantitative measures alone.

**Conclusions:**

Adaptive mechanisms represent legitimate and necessary sustainability strategies in resource-constrained settings, not implementation failures. We propose ‘functional fidelity’ (maintaining core outcomes through flexible processes) and ‘adaptive capacity’ as crucial theoretical extensions for implementation science in LMICs. Sustainable mental health integration requires frameworks that recognise and support frontline innovation while ensuring quality safeguards are maintained, offering a more realistic pathway to closing the global mental health treatment gap.

Mental health disorders impose a significant global burden, affecting over 970 million people worldwide, with a disproportionate impact in low- and middle-income countries (LMICs) where approximately 85% of individuals with severe mental illness receive no effective care [1,2]. Sub-Saharan Africa exemplifies this crisis, with mental health conditions contributing nearly 20% of the total disease burden, while receiving less than 1% of health budgets [3]. Nigeria starkly illustrates these disparities: an estimated 64 million people experience some mental health condition, yet fewer than 300 specialists serve the country’s whole population, which exceeds 200 million [4,5]. The World Health Organization's Mental Health Gap Action Programme advocates for integrating mental health services into primary healthcare through task-shifting approaches, training non-specialist providers to deliver evidence-based interventions [6]. While initial integration efforts demonstrate feasibility and clinical effectiveness of said interventions, sustaining them at scale remains challenging [7–9]. Many programmes experience ‘programme drift’, a gradual deviation from standardised protocols through local adaptations, and ‘voltage drop’, a reduction in clinical effectiveness when interventions transition from controlled research to routine delivery contexts [10,11].

Current sustainability research relies heavily on frameworks developed in high-income settings, including the Organizational Readiness to Change Assessment (ORCA) and the NHS Sustainability Index (NHS-SI), which identify critical predictors of sustainability such as leadership capacity and resource availability [12,13]. However, these quantitative approaches often fail to explain why certain strategies succeed or fail in resource-constrained settings, as well as how frontline actors develop innovative responses to chronic underfunding, high staff turnover, and community stigma [14]. This explanatory gap is particularly acute in LMICs, where the dynamic tension between maintaining intervention fidelity and adapting to local realities remains under-theorised [15].

Emerging scholarship suggests that some programme drift, such as peer mentoring networks that compensate for formal training gaps, might represent constructive modifications that enhance intervention appropriateness rather than implementation failures [16,17]. Similarly, voltage drop could prompt local solutions that preserve essential intervention components despite reduced effectiveness [18]. Mixed-methods designs offer unique advantages for understanding these sustainability dynamics, combining the quantitative validation of predictors with qualitative depth in order to better understand organisational processes and stakeholder experiences [19]. Ethnographic methods are particularly useful for exploring adaptive mechanisms and stakeholder-developed solutions that remain invisible to standardised quantitative tools [20,21].

Lagos State’s Mental Health in Primary Care (MeHPriC) programme provides an exceptional case for examining sustainability dynamics in practice [22–24]. Serving over 20 million residents with an estimated 12% prevalence of common mental disorders [25], Lagos launched MeHPriC in 2013 to integrate mental health services into primary health care through Mental Health Gap Action Programme-guided task-shifting and technology support, targeting depression, psychosis, epilepsy, and substance use disorders. Following a successful pilot phase that began with less than 10% primary health centre coverage, MeHPriC was scaled to 57 flagship facilities by 2017, achieving 70% geographic coverage and training over 800 non-specialist providers [26].

However, sustainability challenges emerged soon after implementation: a 30% annual provider turnover disrupted protocol adherence, medication stockouts affected 30% of facilities monthly and compromised treatment continuity, while community stigma deterred an estimated 40% of eligible patients from seeking care [26]. These conditions created natural variation in programme drift and voltage drop, providing opportunities to examine when and how adaptive mechanisms emerge.

This investigation makes three novel contributions to implementation science. First, we provide the first empirical evidence that certain forms of programme drift function as legitimate sustainability mechanisms in LMICs, challenging dominant fidelity-focussed frameworks. We introduce the concept of ‘functional fidelity’, defined as maintaining core intervention outcomes, while allowing process flexibility, as an alternative to rigid protocol adherence. Second, we demonstrate how mixed-methods approaches can reveal implementation dynamics invisible to either quantitative or qualitative methods alone, particularly in terms of adaptive mechanisms that standard sustainability assessments fail to capture. Third, we extend the Integrated Sustainability Framework (ISF) [10] by proposing ‘adaptive capacity’ as a new domain for resource-constrained settings, with operational criteria for distinguishing constructive adaptations that enhance sustainability from harmful deviations that compromise care quality.

Our specific objectives are to: identify multi-level barriers and facilitators to MeHPriC sustainability through stakeholder perspectives and ethnographic observation; examine how programme drift and voltage drop manifest in daily implementation practice and organisational responses; validate qualitative themes through systematic triangulation with quantitative sustainability assessments; and develop contextually grounded recommendations for enhancing sustainability in similar mental health integration efforts in LMICs.

Unlike previous studies that have primarily catalogued implementation barriers [10,27,28], our approach combines the rigorous measurement of sustainability predictors with an in-depth investigation of the adaptive mechanisms through which organisations maintain services despite systemic constraints. While existing sustainability frameworks identify what predicts success, they cannot explain how successful sites operationalise these factors in practice – a gap this study directly addresses. By critically interrogating the contested boundary between fidelity and adaptation, we advance both the science and practice of sustaining equitable mental health care in resource-constrained settings.

## METHODS

### Study design and theoretical framework

This study adopted a convergent parallel mixed-methods design to examine sustainability dynamics within Lagos State’s MeHPriC programme. We collected quantitative and qualitative data simultaneously and analysed them independently before systematically integrating them into our interpretation. This design was selected because while quantitative measures can identify sustainability predictors, qualitative investigation can better reveal the contextual mechanisms explaining how and why these factors operate, particularly regarding adaptive responses to programme drift and voltage drop.

Two theoretical frameworks guided data collection and analysis. The ISF [10] emphasised dynamic interactions between contextual, organisational, and implementation factors, which we extended here to include adaptive and buffering capacity constructs for LMICs. The Supporting the Use of Research Evidence (SURE) [29] framework provided a multi-level analytical structure across health system, organisational, and stakeholder domains.

### Setting and participant selection

We performed research in six local government areas (LGAs) purposively selected from among Lagos State’s 20 LGAs using maximum variation sampling. Selection criteria included: urban/rural classification (urban: >5000/km^2^, peri-urban: 1000–5000/km^2^, rural: <1000/km^2^), MeHPriC implementation duration of ≥18 months, and variation in preliminary administrative performance data showing differences in provider retention rates (range: 45–85% annually) and service utilisation (range: 15–45 patients/month per facility). The selected LGAs were Lagos Island and Surulere (urban), Alimosho and Agege (peri-urban), and Badagry and Epe (rural).

We determined a target of 130 participants pragmatically based on available stakeholders across selected sites and comparable LMIC mixed-methods studies. A *post-hoc* power analysis indicated 80% power to detect medium effect sizes (Cohen’s d = 0.5) for key predictors. To partially address potential bias in our convenience sampling approach, we employed snowball sampling specifically to identify disengaged users and critical perspectives, including three care recipients who had discontinued services and two health workers who had left MeHPriC facilities.

We recruited participants using stratified purposive sampling from among the following groups: policymakers/administrators, such as ministry officials and LGA coordinators; programme managers, such as MeHPriC coordinators and supervisors; health workers, including physicians, nurses, and community health officers from 10 randomly selected primary health centres; and care recipients, comprising patients who had been receiving MeHPriC services within six months. For qualitative data, we selected a nested subsample (n = 70) using maximum variation sampling, including survey participants and snowball sampling, to capture diverse implementation experiences and critical perspectives.

### Data collection procedures

Trained research assistants fluent in English or Yoruba administered four validated instruments: the NHS-SI [13], which measures ten key sustainability determinants, including staff engagement, infrastructure adequacy, and stakeholder involvement (Cronbach’s α = 0.78); the ORCA [12], which evaluates institutional capacity for implementing and sustaining evidence-based practices, assessing leadership characteristics and resource availability (Cronbach’s α = 0.82); the Sustained Implementation Support Scale (SISS) [30], which assesses implementation support mechanisms, particularly supervision quality and training adequacy (Cronbach’s α = 0.85); and the Perceived Intervention Characteristics Scale, a locally developed acceptability and feasibility scale that captures stakeholder perceptions of MeHPriC intervention characteristics across three domains (Cronbach’s α = 0.79). The instruments were targeted by stakeholder relevance: the NHS-SI and ORCA were administered to policymakers, managers, and health workers (n = 80); the SISS to managers and health workers (n = 60); and the Perception Scale to care recipients (n = 50).

Two research fellows (OEO and OO) with an Msc in clinical psychology and five years of qualitative research experience performed in-depth interviews over six months under the supervision of a senior researcher (AOA; PhD in psychiatry, 15 years of qualitative research experience), using semi-structured guides based on the ISF and SURE framework domains. No prior relationships existed between interviewers and participants beyond professional consultation roles. We piloted the semi-structured guides with five non-participant primary health centre (PHC) staff and refined them based on their feedback. Interviews lasted 45–75 minutes (mean (x̄) = 62 minutes) and were conducted in the participants’ preferred language (English or Yoruba) in private settings with only the participant and researcher present. All interviews were audio-recorded with the participants’ full consent and transcribed *verbatim*, with the interviews performed in Yoruba back-translated (semantic equivalence κ = 0.91). We conducted seven repeat interviews for clarification and recorded field notes immediately after each session. Data saturation was achieved by interview 65 and was confirmed through a thematic redundancy analysis, where no new codes emerged in the final five interviews.

Institutional ethnography involved three months of observation across five PHCs representing diverse implementation contexts. AOA and BO conducted 45 clinical sessions and 23 group discussions, documenting workflows, patient-provider interactions, and adaptive mechanisms, as well as completing field notes immediately after each session.

### Data analysis

We used SPSS (IBM Corporation, Armonk, New York, USA), version 28.0 and Stata, version 17.0 (StataCorp LLC, College Station, Texas, USA) to summarise our data descriptively with means and standard deviations for normally distributed continuous variables (verified using Shapiro-Wilk tests), medians and interquartile ranges for non-normal distributions, and frequencies and percentages for categorical variables. We compared groups using independent *t*-tests for continuous variables and χ^2^ tests for categorical variables. We also performed multiple linear regression to examine sustainability predictors using NHS-SI total scores as the dependent variable, while selecting covariates based on theoretical relevance and bivariate associations (*P* < 0.20 threshold). Model assumptions were verified for normality (Shapiro-Wilk *P* = 0.18), homoscedasticity (Breusch-Pagan *P* = 0.31), linearity (visual inspection of scatterplots), and multicollinearity (all variance inflation factors (VIFs) <2.5). We reported both unadjusted and adjusted effect estimates with 95% confidence intervals.

We performed a hybrid thematic analysis in NVivo 12 (QSR International, Burlington, Massachusetts, USA) that combined deductive coding from the ISF and SURE frameworks with inductive theme development. Two trained researchers (EA and SA) with five years of qualitative research experience independently coded 20% of the transcripts, achieving an inter-coder reliability of κ = 0.88. Disagreements were resolved through consensus with a third reviewer (BO). We also used comparative analysis to examine patterns across stakeholder groups and facility contexts.

Systematic triangulation identified convergent (robust evidence) and divergent findings (explored through additional analysis), as well as complementary insights using joint displays and side-by-side matrices. Meta-inferences combined quantitative predictors with qualitative mechanisms to explain sustainability dynamics.

### Quality assurance and ethical considerations

We obtained ethics approval from the Lagos State University Teaching Hospital Ethics Committee (LREC/06/10/1443). Written informed consent was secured from all participants, with ongoing consent procedures utilised during the ethnography. Confidentiality protections included data anonymisation, encrypted storage, and aggregate-only reporting, while maintaining sufficient descriptive context for analysis. Bias mitigation strategies included using a diverse research team, pilot testing all instruments, including disengaged users, systematically documenting non-response patterns, and holding regular debriefing sessions. Five percent of surveys and 10% of transcripts were independently audited for quality assurance.

## RESULTS

### Participant characteristics and response patterns

A total of 130 stakeholders completed the quantitative surveys (96.3% response rate) and 70 participated in qualitative interviews (93.3% response rate), with ethnographic observations spanning 45 sessions across five PHCs. Non-response was minimal, with five individuals declining quantitative participation (due to time constraints, n = 3, or privacy concerns, n = 2) and five having scheduling conflicts for qualitative interviews; there were no systematic differences by available characteristics. Participant characteristics show diverse representation across policymakers/administrators (n = 20; mean experience of 12.3 years, standard deviation (SD) = 3.1), programme managers (n = 10; mean involvement of 3.2 years, SD = 1.2), health workers (n = 50, 60% female, median experience of two years, interquartile range = 1–4), and care recipients (n = 50, primarily with depression, 42%, and epilepsy, 28%). Health workers had an average of 8.2 years of primary care experience, while care recipients averaged 2.3 years since their first MeHPriC contact (**Table 1**).

**Table 1:** Participant characteristics and mixed-methods data collection summary*

	Quantitative (n = 130)	Qualitative (n = 70)	Ethnographic (n = 5 PHCs)
**Response rates**			
Invited	135	75	
Participated	130 (96.3; 92.1, 98.7)	70 (93.3; 85.1, 97.8)	45 sessions, 23 group discussions
Non-response	Time constraints (n = 3); privacy concerns (n = 2)	Scheduling conflicts (n = 5)	
**Stakeholder distribution, n (%)**			
Policymakers/administrators	20 (15.4)	10 (14.3)	
Programme managers	10 (7.7)	10 (14.3)	
Health workers	50 (38.5)	30 (42.9)	All facilities
Care recipients	50 (38.5)	20 (28.6)	Waiting areas observed
**Key demographics**			
Age in years, x̄ (SD)	41.2 (12.3)	39.8 (11.7)	
Female	78 (60.0; 51.2, 68.4)	43 (61.4; 49.2, 72.6)	
Urban residence	52 (40.0; 31.6, 48.9)	28 (40.0; 28.5, 52.4)	3 facilities
**Professional experience**			
Primary care in years	8.2 (5.4)	8.7 (5.9)	Range: 2–15 y
MeHPriC involvement in years, MD (IQR)	2.0 (1.0–4.0)	2.0 (1.0–4.0)	Range: 1.5–4.2 y
**Care recipient diagnoses**			
Depression	21 (42.0; 28.2, 56.8)	8 (40.0; 19.1, 63.9)	Most common presentation
Epilepsy	14 (28.0; 16.2, 42.5)	6 (30.0; 11.9, 54.3)	Monthly clinic attendance
Psychosis	9 (18.0; 8.6, 31.4)	4 (20.0; 5.7, 43.7)	Often family-accompanied
**Data collection quality**			
Saturation achieved		Interview (n = 65)	Thematic redundancy
Inter-coder reliability, κ (95% CI)		0.88 (0.82–0.94)	Field note consensus
Missing data	<2% per variable	None	Daily completion

CI – confidence interval, IQR – interquartile range, MD – median, PHC – primary health center, SD – standard deviation, x̄ – mean

*Values are presented as n (%; 95% confidence interval) unless specified otherwise.

### Multi-level sustainability barriers and adaptive responses

#### System-level constraints drive programme drift and voltage drop

Systematic underfunding emerged as the primary barrier. Mental health received less than 2% of Lagos State’s health budget, causing medication stockouts in 30% of facilities monthly (**Table 2**). The NHS-SI infrastructure scores (x̄ = 2.7/5, SD = 0.9) validated these resource constraints, while ethnographic observations documented treatment interruptions that compromised clinical effectiveness, thereby creating a ‘voltage drop’. A programme manager explained: ‘Without steady funding, we’re constantly firefighting stockouts, which undermines patient trust and clinical outcomes.’

**Table 2:** Multi-level sustainability barriers and facilitators with quantitative validation*

SURE level	Key barriers	Key facilitators	Quantitative evidence	Representative quote
Health system – resource constraints	Systematic underfunding (<2% budget); medication stockouts (30% monthly); infrastructure failures	Mental health desk coordination; non-governmental organisation partnerships ( ~ 10% funding); policy advocacy	NHS-SI infrastructure: x̄ (SD) = 2.7 (0.9); β (95% CI) = −0.35 (−0.50, −0.20; *P <* 0.01. Funding adequacy: x̄ (SD) = 2.4 (1.0).	‘Without steady funding, we're constantly firefighting stockouts, undermining patient trust’ – manager
Organisational – workforce issues	High turnover (30% annually); skill gaps; competing priorities	Peer mentoring networks; adaptive local leadership; role flexibility	NHS-SI leadership: x̄ (SD) = 3.9 (0.6); β (95% CI) = 0.42 (0.29, 0.55; *P* < 0.001 . SISS training: x̄ (SD) = 2.9 (0.8); r = –0.31; *P* < 0.01 with turnover.	‘We teach new staff ourselves: it works better than waiting for official training’ – CHO
**Individual/community – access barriers**	Community stigma (40% deterrence); transportation costs; provider burnout	High intervention acceptability; peer support networks; intrinsic motivation	Perception feasibility: x̄ (SD) = 3.1 (0.8). Acceptability: x̄ (SD) = 4.2 (0.5). Stigma: β = −0.30 (−0.45, −0.15; *P* < 0.05).	‘Neighbors call me “mad woman” (…) I skip appointments to avoid judgment’ – patient

CHO – community health officer, CI – confidence interval, NHS-SI – NHS Sustainability Index, r – Pearson correlation coefficient, SD – standard deviation, SISS – Sustained Implementation Support Scale, SURE – Supporting Use of Research Evidence, x̄ – mean, β – correlation coefficient

*Model statistics: R^2^ = 0.45, F(5,74) = 12.1, *P* < 0.001.

Workforce instability compounded system challenges, with a 30% annual staff turnover disrupting protocol adherence and contributing to programme drift as new staff developed *ad hoc* practices. However, the mental health desk provided critical mitigation through policy coordination and partnerships with non-governmental organisations, securing approximately 10% of operational funding, while maintaining programme visibility.

#### Organisational innovation through adaptive mechanisms

Rather than viewing programme drift as failure, our analysis revealed adaptive mechanisms that preserved service delivery despite constraints. Peer mentoring networks developed organically in four of the five ethnographic sites, where experienced staff systematically oriented newcomers to mental health protocols. These informal systems often exceeded official supervision in frequency and practical relevance. A senior nurse described: ‘When new staff arrive, I spend time showing them the mental health protocols. It's not official, but it works better than waiting for formal training.’

Role flexibility emerged as another adaptive strategy, with pharmacy assistants providing medication counselling and data clerks conducting initial screenings during staffing shortages. While deviating from formal protocols, these practices demonstrated organisational responsiveness and prevented service discontinuation. Simplified documentation procedures developed locally reduced the administrative burden while maintaining essential clinical records, exemplifying ‘functional fidelity’, which preserves intervention outcomes through flexible processes. The NHS-SI leadership scores (x̄ = 3.9/5, SD = 0.6) reflected recognition of these adaptive efforts, with facility heads developing resource-sharing arrangements and rotating supervision schedules despite limited specialist availability.

#### Individual-level barriers and community dynamics

Community stigma deterred an estimated 40% of eligible patients, contributing to voltage drop through reduced service uptake. Perception Scale feasibility scores (x̄ = 3.1/5, SD = 0.8) confirmed these access barriers, while ethnographic observations documented patients avoiding clinics due to fears of social labeling. A patient with depression explained: ‘My neighbours call me 'the mad woman' when they see me at the clinic. Sometimes I skip appointments to avoid their judgment.’

Provider skill gaps resulting from inadequate training (SISS scores: x̄ = 2.9/5, SD = 0.8) led to simplified assessments and inappropriate referrals. However, high intervention acceptability (x̄ = 4.2/5, SD = 0.5) sustained provider motivation, with health workers expressing deep satisfaction from seeing patient improvement: ‘Seeing someone who was tied up at home now coming to the clinic dressed and calm gives me energy to continue despite everything.’

### Quantitative validation and mixed-methods triangulation

#### Regression analysis results

Systematic triangulation showed strong convergence between quantitative predictors and qualitative themes (**Table 3**). Multiple regression analysis confirmed key qualitative themes, explaining 45% of the variance in sustainability (R^2^ = 0.45, F(5,74) = 12.1, *P* < 0.001). After adjustment for covariates, the most significant predictors were infrastructure adequacy (*β* = −0.35, *P* < 0.01), leadership quality (*β* = 0.42, *P* < 0.001), and community stigma (*β* = −0.30, *P* < 0.05). These quantitative findings aligned closely with the qualitative data; for instance, low infrastructure scores corroborated narratives of stockouts, and stigma's negative coefficient supported reports of 40% patient deterrence.

**Table 3:** Complete regression analysis – unadjusted and adjusted models*

	Unadjusted model	Adjusted model†


**Predictor**

**β (95% CI)**

***P*-value**

Infrastructure adequacy

−0.42 (−0.58, −0.26)

<0.001

Leadership quality

0.39 (0.22, 0.56)

<0.001

Community stigma

−0.33 (−0.49, −0.17)

<0.001

Training adequacy

−0.28 (−0.44, −0.12)

0.003

Intervention acceptability

0.25 (0.08, 0.42)

0.012

**Model statistics**

R^2^

0.38

F-statistic

F(4,75) = 11.6

<0.001

AIC

342.1

BIC

353.4

AIC – Akaike information criterion, BIC – Bayesian information criterion, CI – confidence interval, β – correlation coefficient

*Model diagnostics: normality – Shapiro-Wilk *W* = 0.981, *P* = 0.18; homoscedasticity – Breusch-Pagan χ^2^ = 7.32, *P* = 0.31; linearity – confirmed via residual plots (available upon request); multicollinearity – all variance inflation factors <2.5 (range: 1.2–2.4). Dependent variable: NHS-SI total sustainability score (range: 0–50). Missing data: <2% per variable, handled via listwise deletion. n = 80 for unadjusted models; n = 79 for adjusted models due to missing covariate data

†Adjusted for facility size, urban/rural location, implementation duration, and baseline performance scores.

#### Stakeholder priority divergences

There were systematic divergences in stakeholder priorities, with policymakers focused on funding, health workers on operational challenges, and care recipients on access barriers, which explains why single-level interventions often fail and underscores the need for coordinated, multi-level approaches (Table S1 in the **Online Supplementary Document**).

#### Mixed-methods integration reveals hidden dynamics

**Figure 1:**
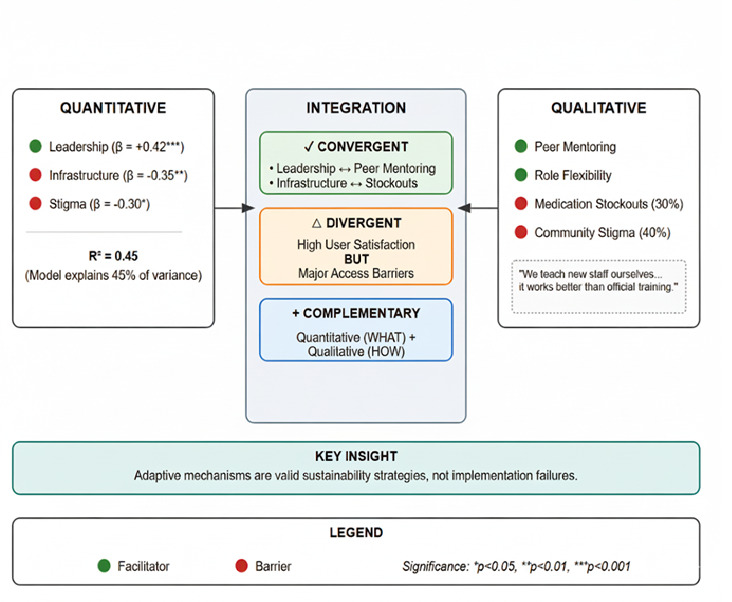
Mixed-methods integration of findings.

The mixed-methods approach brought unique value to our analysis (**Figure 1**). The quantitative analysis identified leadership as the strongest positive predictor of sustainability, but could not explain what effective leadership entailed in practice. Ethnographic observation revealed that this operated through informal peer mentoring networks, a mechanism invisible to standardised scales but critical for maintaining services despite high staff turnover. The integration also uncovered some contradictions. While care recipients who accessed services reported high satisfaction (x̄ = 4.2/5), this positive metric masked significant population-level access barriers due to stigma, highlighting that relying solely on user satisfaction can provide a misleading assessment of a programme's overall sustainability and reach. The moderate explanatory power of the regression model (R^2^ = 0.45) further showed that the adaptive mechanisms observed qualitatively constitute a crucial, yet often unmeasured, domain in sustainability.

#### Data quality and robustness

Missing data were minimal (<2% per variable), with no systematic patterns (Little’s test for missing completely at random *P* = 0.43). All regression assumptions were satisfied: normality (Shapiro-Wilk *P* = 0.18), homoscedasticity (Breusch-Pagan *P* = 0.31), and multicollinearity (VIF range = 1.2–2.4). Sensitivity analyses confirmed the robustness of key findings across multiple model specifications.

The moderate R^2^ of 0.45 indicates substantial unmeasured variance, suggesting that additional contextual factors merit investigation. The model assumptions were satisfied and multicollinearity was acceptable (VIF <2.5).

## DISCUSSION

### Adaptive mechanisms as legitimate sustainability strategies

This mixed-methods investigation provides empirical evidence that adaptive mechanisms function as legitimate sustainability strategies, rather than as implementation failures in mental health integration in LMICs. Our findings fundamentally challenge implementation science frameworks that view programme drift and voltage drop solely as threats to intervention fidelity. Peer mentoring networks that emerged organically maintained protocol knowledge, despite a 30% annual turnover, directly countering programme drift. Role flexibility enabled service continuity during staffing shortages, while simplified documentation preserved essential clinical information without imposing an excessive administrative burden.

These adaptations demonstrated ‘functional fidelity’, maintaining core intervention outcomes through flexible implementation processes – a concept with profound implications for sustainability planning in resource-constrained settings. However, reframing programme drift as adaptive carries significant risks, as not all adaptations enhance sustainability. Our analysis suggests three empirically grounded criteria for distinguishing constructive from harmful adaptations: maintaining clinical effectiveness and patient safety outcomes; addressing genuine resource constraints rather than convenience; and incorporating systematic stakeholder input and quality monitoring. In essence, ‘functional fidelity’ is achieved when adaptations satisfy these three core conditions, ensuring that flexibility enhances rather than compromises care quality. Without these safeguards, ‘adaptive’ could become a euphemism for substandard care or system underinvestment.

### Extending implementation science theory to LMICs

**Figure 2:**
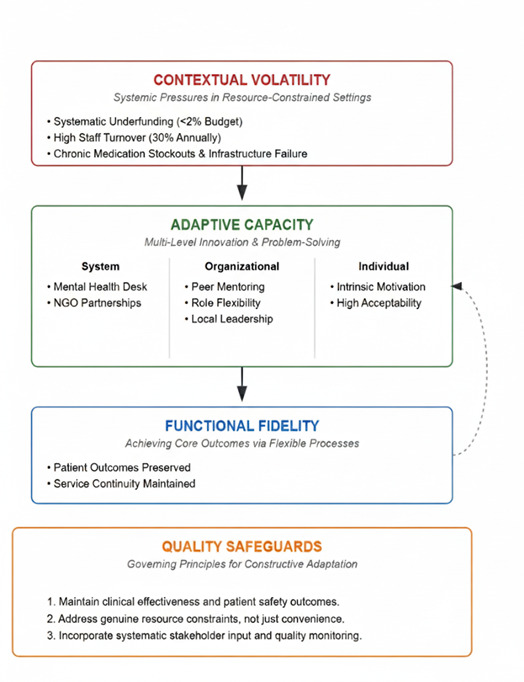
Adaptive sustainability framework, proposed for LMIC mental health integration.

Our core theoretical contribution involves extending the ISF through three specific additions validated by our empirical findings (**Figure 2**). First, ‘adaptive capacity’ should be recognised as a distinct sustainability domain encompassing organisational learning, a problem-solving culture, and tolerance for beneficial protocol modifications. Unlike traditional resource adequacy measures, adaptive capacity explains how some sites succeed despite constraints while others with similar resources fail. Second, ‘contextual volatility’ requires explicit assessment in LMIC frameworks, including resource predictability, workforce stability, and infrastructure reliability. Lagos’ systematic underinvestment and high turnover exemplify this volatility's impact on sustainability. Third, ‘functional fidelity’ offers an alternative to rigid protocol adherence, prioritising outcome achievement over process compliance when implementation contexts differ markedly from intervention development settings. This concept bridges the tension between evidence-based practice and contextual responsiveness.

### Methodological insights from mixed-methods integration

A key contribution of this study is its demonstration of how mixed-methods research can reveal implementation dynamics that are invisible to any single approach. As detailed above, our integrated analysis moved beyond simply identifying what predicts sustainability to explain how those predictors function in practice. For example, we uncovered the specific adaptive mechanisms, like peer mentoring, that operationalised the statistically significant finding of strong leadership. Furthermore, the triangulation process was essential for contextualising quantitative data. By juxtaposing high user satisfaction ratings with pervasive community stigma, our approach highlighted the risk of drawing incomplete conclusions from user-only metrics. This shows that understanding the tension between fidelity and adaptation requires the depth that mixed-methods designs provide.

### Multi-level policy implications

Our findings suggest several multi-level policy implications. In terms of immediate strategies (0–6 months), policies should strengthen existing peer mentoring by officially allocating protected time and structured curricula, and should address high staff turnover through modest retention incentives and career advancement opportunities. At the medium-term (6–18 months), interventions should focus on establishing medication buffer stocks to reduce stockouts, as well as implementing community-led stigma reduction campaigns to improve patient access. Lastly, when considering long-term system strengthening (≥18 months), policies should scale the mental health desk model to local government levels, and advocate systematically for a 5% mental health budget allocation through evidence-based policy briefs. Successfully implementing these long-term strategies, particularly securing budget increases, will require navigating significant political hurdles and demonstrating a clear return on investment to policymakers. Critical quality assurance, including systematic patient outcome monitoring, must be embedded to prevent adaptive mechanisms from masking systemic neglect. We summarise these multi-level intervention strategies with specific timelines for implementation across different stakeholder priorities in Table S2 in the **Online Supplementary Document****.**

### Transferability to similar LMIC settings

Our findings, though specific to Lagos, offer insights relevant to other LMICs facing similar constraints. The adaptive mechanisms documented, including peer mentoring and role flexibility, likely represent generalisable responses to the resource scarcity and workforce limitations common across sub-Saharan Africa [3]. The multi-level nature of sustainability barriers suggests that single-factor interventions may have limited impact without addressing interconnected system constraints. However, specific manifestations will require contextual adaptation, acknowledging local differences in health systems and cultural factors.

### Study limitations and future research directions

Our study has several limitations. The cross-sectional design limits causal inferences, though the programme’s maturity enabled observation of later-stage challenges. High response rates may reflect a bias toward engaged stakeholders and could be influenced by social desirability bias, where participants might overstate positive experiences. While we included some disengaged users, our sampling may have missed the most marginalised populations. However, the systematic exclusion of completely disengaged stakeholders remains a limitation, potentially underestimating barriers and overestimating satisfaction levels. This highlights a potential bias in our analysis towards engaged stakeholders.

For our quantitative analysis, we note that while our *post-hoc* power analysis indicated 80% power to detect medium effect sizes (Cohen's d = 0.5) for key predictors, we acknowledge the absence of formal power calculations as a limitation. The limitations of the regression analysis include a modest sample size when stratified by stakeholder groups, which could potentially limit the power to detect smaller effects. Despite the model assumptions being satisfied and multicollinearity being acceptable, the findings should be interpreted as exploratory rather than definitive, given the modest sample size, cross-sectional design and convenience sampling approach. This further reinforces the need for qualitative investigation to capture the full picture of sustainability in complex systems.

The ethnographic component, while providing rich insights, may have been influenced by the observer effect (Hawthorne effect), where the presence of researchers could have altered the natural behaviours of staff and patients. Furthermore, the geographic focus on Lagos may limit generalisability; as a large urban megacity, its health system and resource dynamics may not represent those of more rural or conflict-affected LMICs. Future research should include longitudinal studies, intervention trials to test beneficial adaptations, and multi-country comparative analyses.

## CONCLUSIONS

This study fundamentally reframes sustainability in mental health integration in LMICs, demonstrating that adaptive mechanisms represent legitimate strategies for maintaining services despite systemic constraints. Through rigorous mixed-methods investigation, we show that peer mentoring networks, role flexibility, and functional fidelity can preserve intervention effectiveness while enhancing local appropriateness, challenging fidelity-focussed frameworks that may be unsuitable for resource-constrained settings. Our theoretical contributions of adaptive capacity, contextual volatility, and functional fidelity extend the ISF to better reflect realities of LMICs. For policy and practice, these findings advocate for strategies that support beneficial adaptations while maintaining quality safeguards, offering a more realistic and context-sensitive pathway toward closing mental health treatment gaps

## Additional material


Online Supplementary Document
