# Supplementary material for: Exploring contextual barriers and facilitators to sustaining mental health integration in primary care: a mixed-methods analysis of adaptive mechanisms and multi-level dynamics in Lagos, Nigeria
Source: J Glob Health. 2025 Nov 7;15:04305. doi: 10.7189/jogh.15.04305 (PMC12593173; doi:10.7189/jogh.15.04305)
Supplement: Online Supplementary Document [file jogh-15-04305-s001.pdf]

Supplement to: Adewuya A, Ola B, Abimbola S, Abdulmalik J. Exploring contextual barriers and facilitators to sustaining mental health integration in primary care: a mixed-methods analysis of adaptive mechanisms and multi-level dynamics in Lagos, Nigeria. J Glob Health. 2025;15:04305.

Table S1. Mixed-Methods Triangulation Matrix

Sustainability Domain	Quantitative Evidence	Qualitative Evidence	Integration Type	Meta-Inference
**CONVERGENT FINDINGS**				
**Resource Constraints**	NHS-SI Infrastructure: M=2.7±0.9 β=-0.35** (-0.50, -0.20)	30% monthly stockouts documented Treatment interruptions observed	**Strong Convergence**	Resource scarcity creates voltage drop through service disruption
**Adaptive Leadership**	NHS-SI Leadership: M=3.9±0.6 β=0.42*** (0.29, 0.55)	Peer mentoring networks Problem-solving culture observed	**Strong Convergence**	Leadership operates through adaptive mechanisms invisible to standard measures
**Community Stigma**	Stigma β=-0.30* (-0.45, -0.15) Feasibility M=3.1±0.8	40% patient deterrence Social labeling fears documented	**Strong Convergence**	Multi-level stigma barriers require community-level interventions
**DIVERGENT FINDINGS**				
**Service Experience**	High acceptability: M=4.2±0.5 Strong predictor of retention	Significant access barriers Transport costs, scheduling issues	**Explanatory Divergence**	Satisfaction among engaged users ≠ population accessibility
**Training Adequacy**	Moderate SISS scores: M=2.9±0.8 Negative correlation with outcomes	Extensive skill gap concerns Informal peer systems compensate	**Complementary Divergence**	Training frequency ≠ quality; adaptive mechanisms fill gaps
**COMPLEMENTARY INSIGHTS**				
**Sustainability Mechanisms**	Statistical predictors identify **WHAT** factors matter (R^2=0.45)	Ethnographic observation reveals **HOW** mechanisms operate	**Methodological Complementarity**	Quantitative + qualitative understanding exceeds either alone

*Integration reveals both predictors AND processes essential for comprehensive sustainability understanding. Significance: *p<0.05, **p<0.01, ***p<0.001.*

Table S2. Stakeholder Priority Divergences and Multi-Level Intervention Framework

**Stakeholder Group**	**Primary Sustainability Focus**	**Representative Priority**	**Immediate Interventions (0-6 months)**	**Long-term Strategies (12+ months)**
**Policymakers (n=10)**	Structural funding and governance	*"Everything depends on adequate budget allocation and policy support"*	• Advocate 5% mental health budget allocation<br>• Strengthen governance mechanisms	• Scale mental health desk to LGAs<br>• Performance-based financing
**Programme Managers (n=10)**	Organizational coordination challenges	*"Turnover disrupts everything; we need retention and handover systems"*	• Formalize peer mentoring<br>• Retention bonuses	• Career progression pathways<br>• Leadership development programs
**Health Workers (n=30)**	Operational support and capacity	*"We're stretched thin; need training updates and workload relief"*	• Protected mentoring time (2hrs/week)<br>• Refresher training modules	• Workload redistribution<br>• Specialization recognition
**Care Recipients (n=20)**	Access and community acceptance	*"Transport costs more than daily earnings; stigma keeps others away"*	• Community leader partnerships<br>• Radio campaign launch	• Transport subsidies<br>• Peer ambassador programs
**COORDINATED APPROACH**	**Multi-Level Integration Required**	**Systems Thinking Essential**	**Phase 1: Foundation Building**	**Phase 2: Sustainable Systems**
**Cross-cutting needs**	• Resource adequacy (all groups)<br>• Quality maintenance<br>• Community engagement	• No single-level solutions sufficient<br>• Stakeholder alignment critical	• Immediate gap filling<br>• Organic innovation support	• Structural reform<br>• Institutionalized adaptation

Success Metrics by Phase:

- *Phase 1 (6 months)*: Reduce stockouts to <10%, decrease new staff adaptation time to 6-8 weeks, establish mentoring in 80% of facilities
- *Phase 2 (18 months)*: Achieve 5% budget allocation, reduce patient deterrence to <25%, maintain provider retention >85%
